# Mapping quality of life in Norway: psychometric evaluation and network analysis of 15,148 responses from a public health study

**DOI:** 10.7717/peerj.20529

**Published:** 2026-01-13

**Authors:** John Roger Andersen, Tone Nygaard Flølo, Kari Hanne Gjeilo, Käthe Meyer, Tone Merete Norekvål, Gudrun Rohde

**Affiliations:** 1 Department of Health and Caring Sciences, Western Norway University of Applied Sciences, Førde, Norway; 2 Health Research Sogn og Fjordane, Førde Hospital Trust, Førde, Norway; 3 Voss Hospital, Haukeland University Hospital, Voss, Norway; 4 Department of Nursing and Health Promotion, Oslo Metropolitan University, Oslo, Norway; 5 Department of Public Health and Nursing, Faculty of Medicine and Health Sciences, NTNU—Norwegian University of Science and Technology, Trondheim, Norway; 6 Department of Cardiology, St. Olav’s Hospital, Trondheim University Hospital, Trondheim, Norway; 7 Oslo University Hospital, Oslo University Hospital, Oslo, Norway; 8 Department of Heart Disease, Haukeland University Hospital, Bergen, Norway; 9 Department of Health and Caring Sciences, Western Norway University of Applied Sciences, Bergen, Norway; 10 Centre on Patient-Reported Outcomes, Haukeland University Hospital, Bergen, Norway; 11 University of Bergen, Bergen, Department of Clinical Science, Norway; 12 Department of Health and Nursing, University of Agder, Kristiansand, Norway; 13 Department of Clinical Research, Sørlandet Hospital, Kristiansand, Norway

**Keywords:** Self-reported health, Quality of life, Psychometric properties, Public health, Network analysis

## Abstract

**Background:**

The Norwegian Quality of Life Study (NQoLS) was established to inform public health policy by assessing self-reported health (SRH) and quality of life (QoL) outcomes across the general population, identifying factors that influence these outcomes, and highlighting vulnerable groups. In this study, we assessed the psychometric properties of the NQoLS measures and applied network analysis to explore the structural relationships among outcome variables.

**Methods:**

The 2022 NQoLS is a cross-sectional study that included 15,148 adults from the general adult Norwegian population. No exclusion criteria were specified beyond the requirement that participants have a registered address, email, and/or phone number. The study assessed SRH and QoL through single- and multi-item measures across physical, psychological, and social domains. The psychometric evaluation included descriptive statistics, reliability testing, confirmatory factor analysis, and correlation analysis, followed by a network analysis to map how outcomes connect and cluster.

**Results:**

Measures generally demonstrated acceptable reliability and validity. Model fit for multi-item scales was generally adequate following minor modifications. Network analysis identified a central cluster, including the Satisfaction with life scale, Hopkins Symptom Checklist-5, and Satisfaction with psychological health. These indicators were highly connected and structurally central. In contrast, physical health variables, including Satisfaction with physical health, Pain and discomfort, and General health, were more weakly connected and positioned at the network periphery. The sensitivity analysis, stratified by language preference, yielded results consistent with those of the entire sample.

**Conclusions:**

The NQoLS provides a foundation for mapping SRH and QoL. Most measures worked well, though a few could be fine-tuned for a better fit and sensitivity. Based on our assessment and the structure revealed by the network analysis, physical health appears to be relatively underrepresented in the current survey, suggesting a potential area for future enhancement.

## Introduction

Global initiatives such as the World Happiness Report (Helliwell et al., 2024), the OECD Better Life Initiative (Organisation for Economic Co-operation and Development, 2020), and the World Health Organization (World Health Organization, 2012) have emphasized the importance of incorporating self-rated health (SRH) and quality of life (QoL) measures into policy development. In line with such international efforts, the Norwegian Ministry of Health and Care Services mandated the Norwegian Directorate of Health to develop a national strategy for collecting robust population-level SRH and QoL data (Ministry of Health Care Services, 2015). The Norwegian Quality of Life Study (NQoLS) was established in response to this mandate (Nes, Hansen & Barstad, 2018; Pettersen & Engvik, 2022). However, even as interest in SRH and QoL measurement grows globally, methods used in large-scale public health studies remain debated (Kaplan & Hays, 2022; Pequeno et al., 2020). Variability in content and psychometric limitations may complicate cross-group comparisons, weakening the validity of findings. Therefore, there is a need to standardise and optimise SRH and QoL assessments to ensure they are psychometrically sound and suitable for diverse population studies (Kaplan & Hays, 2022).

The NQoLS has explored a wide range of concepts. However, concerns about the including possible underrepresentation of physical health and biological/medical factors in the NQoLS require further attention (Pettersen & Engvik, 2022). Only a small fraction of the general population—approximately one in 20 individuals lives without health issues, as reported by the Global Burden of Disease study (Murray, 2024). Additionally, a third of individuals experience five or more ailments, highlighting the need for comprehensive and broad health outcome measurement. Therefore, it is crucial to have a clear conceptual understanding of health *versus* QoL and its determinants. WHO defines health as “a state of complete physical, mental, and social wellbeing, and not merely the absence of disease or infirmity” (Schramme, 2023). QoL encompasses this broader definition, wellbeing, life satisfaction, happiness, goal attainment, and functional capacity (Fayers & Machin, 2016). The Wilson and Cleary framework has proven valuable due to its clear categorisation of health and QoL outcomes and mapping the main association pathways between them (Wilson & Cleary, 1995). This structured approach could also be beneficial when applied in public health contexts. However, a comprehensive psychometric evaluation of the Norwegian versions of the instruments employed in the NQoLS has not been undertaken. Consequently, uncertainties persist regarding the validity of these measures and the extent to which the assessed outcomes overlap or interrelate. This gap in empirical knowledge challenges the internal coherence of the assessment (Kaplan & Hays, 2022). Addressing these issues is essential to enable rigorous interpretation of findings and to inform future research based on the NQoLS.

Thus, we analysed the pooled data from the NQoLS, irrespective of language version, as the results are intended to be interpreted as one sample when investigating SRH and QoL outcomes. A primary objective was to evaluate the psychometric properties of the included measures. In addition, we applied network analysis to examine the structural relationships among the outcome variables, enabling the detection of clusters and interconnection patterns that provided a nuanced understanding of how outcomes are linked at the population level. To assess the robustness of our findings, we subsequently conducted sensitivity analyses stratified by language version—Norwegian Bokmål, Norwegian Nynorsk, and English—to evaluate the stability of results.

## Material & Methods

The description of the cross-sectional study design, sample, survey procedures, and health and QoL measures draws on documentation from Statistics Norway. Readers interested in a more thorough understanding of the methodology can refer to previous reports (Pettersen & Engvik, 2022; SIKT, 2023) for a more detailed account.

### Study design and data collection

This article utilises anonymised data from the third wave of the NQoLS. The cross-sectional survey was carried out during 4 weeks, from March 7th to April 6th, 2022. The online survey started with an introduction that explained the purpose of the study and included a consent form. Participants needed to click and agree to fill out the form before proceeding to the questionnaire, so their participation implied their informed consent. Statistics Norway’s survey efforts adhered to the requirements of the Statistics Act and complied with the principles outlined in the EU’s General Data Protection Regulation. The collected data were anonymised and deposited in the Norwegian Agency for Shared Services in Education and Research (SIKT) data repository, freely and openly available for scientific purposes (SIKT, 2023). Utilising anonymised data from the NQoLS does not require ethical approval, as confirmed by the Regional Committee for Medical Research Ethics, South-East Norway (REK no. 712698) (Vederhus et al., 2024).

### Sample

A nationally representative sample of 40,000 individuals aged 18 years and older residing in Norway was randomly selected from the central population database, BeReg, managed by Statistics Norway. The sample was demographically reflective of the Norwegian population. Age was calculated as of March 7th, 2022, the start date for data collection. Participants were contacted through the Contact and Reservation Register, which stores residents’ contact information. The survey was conducted primarily *via* email and SMS, with additional contact made *via* phone where necessary. A small percentage of the sample (0.7%) lacked email addresses, and 7% had no registered mobile numbers. Respondents without electronic contact information were mailed a paper invitation to participate.

### Survey implementation

Data collection was conducted online through a web-based survey. To manage the large sample size, Statistics Norway divided the respondents into 18 dispatch groups, each receiving email and SMS invitations in staggered batches. The survey link was included in these communications, and by completing the questionnaire, participants provided informed consent. Participation was voluntary, and respondents could withdraw at any time. They were assured that their data would be anonymised within 1 year of completion. The questionnaire was available in the two major Norwegian language versions (Bokmål and Nynorsk) and English; the median completion time was 20 min (mean: 24 min). Note that both Nynorsk and Bokmål are mandatory to learn in Norwegian schools, and share virtually identical grammar and core vocabulary, differing mainly in word endings and certain stylistic conventions. Additional demographic and socioeconomic data were sourced from various national registers, including those for education, employment, income, and living conditions, to minimise the respondent burden (Pettersen & Engvik, 2022).

### Measures of health and quality of life

The survey employed well-established, freely available measures recommended by the Norwegian Directorate of Health (Nes, Hansen & Barstad, 2018; Pettersen & Engvik, 2022) to capture multiple facets of SRH and overall QoL outcomes. Details about the study, including the items and scoring of the questionnaire, are available online in an English version (Støren, 2024). An expert group with relevant linguistic and cultural expertise designed and prepared the survey in three languages. Before data collection, the group conducted structured evaluations and user testing to ensure the items were clear and culturally appropriate (Nes, Hansen & Barstad, 2018). Life satisfaction, life meaning, and optimism were assessed using single-item measures, with responses rated on a 0–10 scale, with higher scores indicating better outcomes (Cantril, 1965; Pettersen & Engvik, 2022). General health was rated using a five-point scale ranging from very bad to very good, and Satisfaction with physical or psychological health was rated on a 0–10 scale, with higher scores indicating better outcomes (Pettersen & Engvik, 2022). Pain and discomfort was assessed combined using one item from the EQ-5D-5L, rated on a five-point scale from no pain or discomfort to extreme pain and discomfort (Rabin & De Charro, 2001). In addition to single-item measures, the survey included multi-item scales to assess various dimensions of health and wellbeing. These included the Satisfaction with life scale, where the score ranges from 1 to 7, with higher scores indicating better outcomes (Diener et al., 1985); Hopkins Symptom Checklist-5 (HSCL-5), where the score ranges from 1 to 4, lower scores indicating better outcomes (Strand et al., 2003); Positive and Negative Affect Schedule, where scores ranged from 0 to 10, higher scores indicating more intense effect, either positive or negative (Hansen & Slagsvold, 2012; Watson, Clark & Tellegen, 1988); Pearlin mastery scale, where scores ranged from 1 to 5, higher scores indicating better outcomes (Hysing et al., 2020); European social survey engagement scale, where scores ranged from 0 to 10, higher scores indicating better outcomes (Charalampi, Michalopoulou & Richardson, 2020), and the UCLA loneliness scale, where scores range from 1 to 4, lower scores indicating better outcomes (Steptoe et al., 2013).

### Analysis

We applied a modified version of the Wilson and Cleary framework to categorise variables into (1) biological and physiological variables, (2) symptoms and functioning (which we in this article divide into physical health, psychological health, and social health), (3) general health, and (4) overall QoL (Wilson & Cleary, 1995). In addition, we categorised variables as (5) characteristics of the individual and (6) environmental factors.

Descriptive statistics, including means, medians, standard deviations, skewness, and kurtosis, were calculated to assess data distributions. Skewness ≥2 and kurtosis ≥7 indicated significant deviations from normality, while floor and ceiling effects may be problematic if more than 15% of respondents selected the lowest or highest possible scores. Internal consistency of the scales was evaluated using Cronbach’s alpha, with values ≥0.7 deemed satisfactory (Horney & Tarlov, 1995).

The structural validity of scales with four or more items was assessed using factor analysis (CFA) for ordinal data, employing weighted least squares mean and variance (WLSMV) estimation. This tests whether all items in a scale measure the same underlying concept. Model fit was evaluated using RMSEA, SRMR, CFI, and TLI, with values close to or below .06 for RMSEA, close to or below .08 for SRMR, and close to or above .95 for CFI and TLI considered indicative of good fit (Hu & Bentler, 1999). As these indices capture different aspects of model fit, they should be interpreted together to provide a complementary evaluation of how well the items reflect the intended construct. Importantly, these cutoffs are guidelines rather than strict rules, and model evaluation should also take into account the broader measurement context. Because RMSEA can overestimate misfit when degrees of freedom are small or when categorical indicators are used, its values were interpreted in conjunction with CFI, TLI, and SRMR, which are regarded as more robust under such conditions (Shi, Maydeu-Olivares & Rosseel, 2020).

Correlations between variables were displayed using Pearson correlation coefficients. A regularised partial correlation network was estimated in JASP using the EBICglasso estimator (Bergh van den, 2018; Epskamp et al., 2012; Schönenberg et al., 2023). In the network, each health or QoL measure is shown as a node (a dot), and the lines between them (edges) show how strongly they are related. The correlation structure was determined using JASP’s automatic selection method. Model selection was based on the extended Bayesian Information Criterion (EBIC), with the tuning parameter (γ) set to 0.5. Missing data were handled using listwise exclusion. Network visualisation was generated using edge colour and thickness, reflecting the direction and magnitude of partial correlations. Three standard centrality indices—strength, closeness, and betweenness—were computed and normalised for node comparability. Additionally, the expected influence was estimated to capture net connectivity while preserving the sign of associations. Four alternative metrics—Barrat, Onnela, WS, and Zhang centrality—were also calculated to assess robustness across different centrality frameworks. Two bootstrap analyses tested the network’s stability and accuracy. First, a nonparametric bootstrap assessed edge precision by generating 1,000 resamples and re-estimating the network each time. We compared each edge’s bootstrapped mean and confidence intervals with the original estimates. Narrower intervals indicated greater reliability, whereas wider intervals suggested higher uncertainty. Secondly, a case-dropping bootstrap with 1,000 resamples evaluated the centrality indices (strength, closeness, and betweenness). We progressively removed random portions of the dataset, recalculated each metric, and correlated these with the full-sample results, thus revealing how sensitive each node’s importance was to variations in the sample.

We conducted sensitivity analyses stratified by language version—Norwegian Bokmål, Norwegian Nynorsk, and English—for all tests except the network analysis. In this case, the Nynorsk and English subgroups were too small to support reliable analyses, whereas the Bokmål subgroup was sufficiently large to approximate the pooled analysis closely. Statistical analyses were performed using JASP version 0.95.3 (JASP, 2024).

## Results

A total of 15,148 participants responded to the survey, yielding an overall response rate of 38% (Pettersen & Engvik, 2022; SIKT, 2023). Notably, respondents invited *via* email had a higher participation rate (48%) than those invited by mail (12%). The lowest participation rates were observed among individuals aged 80 and above and those with lower levels of education. Detailed participant demographics are presented in Table 1. Using the modified Wilson and Cleary model, we identified three measures of overall QoL, one for general health, two for physical health, seven for psychological health, and one for social health (Table 2). Additional variables not defined as SRH or overall QoL were categorised into biological/physiological, individual, and environmental characteristics.

**Table 1:** Characteristics of the sample (*n* = 15,148).

Variables	Percentages
Age	
18–24 years	9.0
25–44 years	30.5
45–66 years	41.8
67–79 years	15.9
≥80 years	2.8
Sex	
Female	48.3
Male	51.7
Current marital status	
Not married	37.5
Married	46.4
Other	16.1
Education	
Primary school	13.6
High school	38.1
College/university < 4 years	32.8
College/university ≥ 4 years	15.5
Immigrant status	
Immigrant	11.9
Not immigrant	88.1
County	
Oslo	13.9
Rogaland	8.5
Møre og Romsdal	4.4
Nordland	4.3
Viken	23.8
Innlandet	7.1
Vestfold og Telemark	7.7
Agder	5.6
Vestland	11.5
Trøndelag	8.9
Troms og Finnmark	4.4

**Notes.**

Percentages may not sum to 100 due to rounding.

**Table 2:** Overview and grouping of variables in NQoLS according to an adapted conceptual model by Wilson & Cleary (1995).


**Characteristics of the individual:** gender identity; education; marital status/relationships; living alone or not; number and age of children; health behaviour related to dental care; contribution to others’ happiness; sexual orientation; trust in other people; worries about climate change; life events; work and/or study environment, altruism; self-actualisation; everyday life and free time; health behaviours and economy			
**Biological and physiological variables:** • Age Biological sex	**Physical health:** • Satisfaction with physical health (1 item) • Pain or discomfort (1 item) **Psychological health:** • Optimism (1 item) • Satisfaction with psychological health (1 item) • Hopkins Symptom Checklist-5 (5 items) • Positive emotions (5 items) • Negative emotions (4 items) • Engagement scale (3 items) • Pearlin Mastery Scale (5 items) **Social health:** • UCLA Loneliness Scale (3 items)	**General health:** • General health (1 item)	**Overall quality of life:** • Life satisfaction (1 item) • Life meaning (1 item) • Satisfaction with life scale (5 items)
**Characteristics of the environment:** Housing and local environment; immigrant status; perceptions of society and rights; satisfaction with relation to child/children; satisfaction with partner; satisfaction with friends; the number of people one can rely on if needed; social support; time spent with family and friends; and life events			

**Notes.**

Note: Although the Wilson and Cleary model posits a primarily left-to-right causal flow—from biological to overall Quality of life—bidirectional associations may also emerge in real-world data.

### Psychometric analysis

The measures indicated relatively good health and overall QoL for many participants. Still, the variance in scores also revealed that a significant minority experiences challenges related to SRH and overall QoL (Tables 3–4). Psychometric evaluation of the seven single-item measures generally revealed acceptable measurement properties (Table 3). However, a large ceiling effect was observed for General health and Pain and discomfort. The psychometric analysis of the seven multi-item scales demonstrated acceptable properties, consistent mean and median values, appropriate skewness and kurtosis, minimal floor and ceiling effects, and satisfactory Cronbach’s alpha values (Table 4). CFA conducted on five scales showed acceptable SRMR, CFI, and TLI values. However, four scales exhibited elevated RMSEA values. Modifying covariances between error terms for specific items resulted in improved RMSEA values, rendering them adequate for all scales.

**Table 3:** Descriptive statistics and psychometric properties of single-item measures included in the NQoLS (*n* = 15,148).

Features	Life satisfaction	Life meaning	General health	Satisfaction with physical health	Pain and discomfort	Satisfaction with psychological health	Optimism
Score	0–10, higher score is better	0–10, higher score is better	1–5, lower score is better	0–10, higher score is better	1–5, lower score is better	0–10, higher score is better	0–10, higher score is better
Score range	0–10	0–10	1–5	0–10	1–5	0–10	0–10
Missing data, %	.09	.20	.05	.06	.09	.09	1.34
Mean ± SD	6.86 ± 2.22	7.08 ± 2.20	2.28 ± 0.93	6.34 ± 2.29	2.07 ± 0.94	7.07 ± 2.46	7.38 ± 2.01
Median	7	7	2	7	2	8	8
Skewness	−.68	−.85	.69	−.62	.78	−.89	−1.04
Kurtosis	.10	.53	.16	.05	.30	.20	1.18
% Floor	1.1	1.1	1.7	2.2	1.4	2.0	1
% Ceiling	12.0	13.8	17.8	6.6	29.5	16.7	15.2

**Notes.**

Floor effect, worst possible score; and ceiling effect, best possible score.

**Table 4:** Descriptive statistics and psychometric properties of multi-item scales included in the NQoLS (*n* = 15,148).

Features	Satisfaction with life scale	Hopkins symptom Checklist-5	Positive emotions	Negative emotions	Engagement scale	Pearlin mastery scale	UCLA loneliness scale
Score	1–7, higher score is better	1–4, lower score is better	0–10, higher score is better	0–10, lower score is better	0–10, higher score is better	1–5, higher score is better	1–4, lower score is better
Missing data, %	.32	.17	.26	.21	.07	.03	.13
Mean ± SD	5.09 ± 1.33	1.66 ± 0.67	6.74 ± 1.89	3.47 ± 2.22	6.82 ± 1.87	3.52 ± 0.83	2.24 ± 0.80
Median	5.40	1.40	7.00	3.00	7.00	3.60	2.00
Skewness	−0.87	1.23	−.57	.42	−.77	−.30	.26
Kurtosis	.14	1.17	.15	−.47	.58	−.28	−.66
% Floor	.5	23.5	.3	7.7	.3	.4	11.7
% Ceiling	3.8	.7	4.6	.4	3.4	4.0	3.8
Cronbach’s alpha	.90	.89	.81	.87	.92	.73	.85
RMSEA	.14/.03	.10/.07	.08/.01	.18/.12	NA	.23/.03	NA
SRMR	.02/.00	.03/.01	.01/0.0	.04/.02	NA	.08/.00	NA
CFI	.99/1	.99/1	1/1	.98/.99	NA	.89/.1	NA
TLI	.99/1	.99/1	1/1	.96/.98	NA	.78/1	NA
Modification indices	Item 4*5 (424)	Item c*d (255)	Item 1*7 (56.6)	Item 4*9 (563)	NA	Item 4*5 (2396)	NA

**Notes.**

Floor effect, worst possible score; and ceiling effect, best possible score. RMSEA, root mean square error of approximation; SRMR, standardised root mean square residual; CFI, comparative fit index; TLI, Tucker–Lewis index. NA, not applicable.

Correlational analyses revealed significant associations among the variables (Table 5). As expected, this is consistent with the probable presence of partial conceptual convergence and bidirectional causality.

**Table 5:** Pearson correlations between the outcomes.

Variables		a.	b.	c.	d.	e.	f.	g.	h.	i.	j.	k.	l.	m.	n.
a.	Life satisfaction (1 item)	1	.71	.71	−.47	.47	−.26	.63	.64	−.58	.63	−.48	.56	.41	−.47
b.	Life meaning (1 item)	.71	1	.71	−.47	.47	−.21	.64	.68	−.57	.65	−.44	.67	.39	−.48
c.	Satisfaction with life scale (5 items)	.71	.71	1	−.54	.51	−.28	.64	.63	−.60	.65	−.48	.61	.49	−.54
d.	General health (1 item)	−.47	−.47	−.54	1	−.73	.49	−.51	−.46	.44	−.45	.35	−.39	−.45	−.34
e.	Satisfaction with physical health (1 item)	.47	.47	.51	−.73	1	−.44	.51	.45	−.39	.45	−.32	.43	.38	−.33
f.	Pain and discomfort (1 item)	−.26	-.21	−.28	.49	−.44	1	−.22	-.25	.26	−.21	.20	−.17	−.30	.18
g.	Satisfaction with psychological health (1 item)	.63	.64	.64	−.51	.51	−.22	1	.53	−.74	.66	−.59	.57	.38	−.54
h.	Optimism (1 item)	.64	.68	.63	−.46	.45	−.25	.53	1	−.45	.58	−.34	.57	.50	−.40
i.	Hopkins symptom checklist (5 items)	−.58	−.57	−.60	.44	−.39	.26	−.74	−.45	1	−.59	.74	−.48	−.40	.56
j.	Positive emotions	.63	.65	.65	−.45	.45	−.21	.66	.59	−.60	1	−.48	.65	.42	−.49
k.	Negative emotions	−.48	−.44	−.48	.35	−.32	−.20	−.59	−.34	.73	−.48	1	−.37	−.34	.48
l.	Engagement scale	.56	.67	.61	−.39	.43	−.17	.57	.57	−.48	.65	−.37	1	.37	−.41
m.	Pearlin mastery scale	.41	.39	.49	−.45	.38	−.30	.38	.50	−.40	.42	−.34	.37	1	−.34
n.	UCLA loneliness scale (3 items)	−.48	−.48	−.54	.34	−.33	.18	−.54	−.40	.56	−.49	.48	−.41	−.34	1

### Network analysis

**Figure 1:**
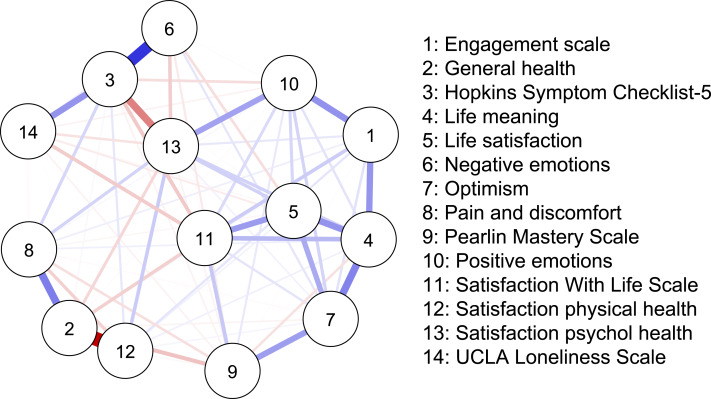
Network structure.

**Figure 2:**
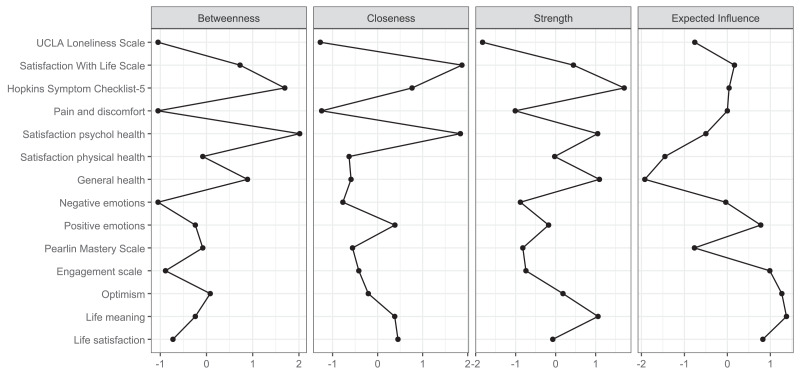
Centrality plot.

**Figure 3:**
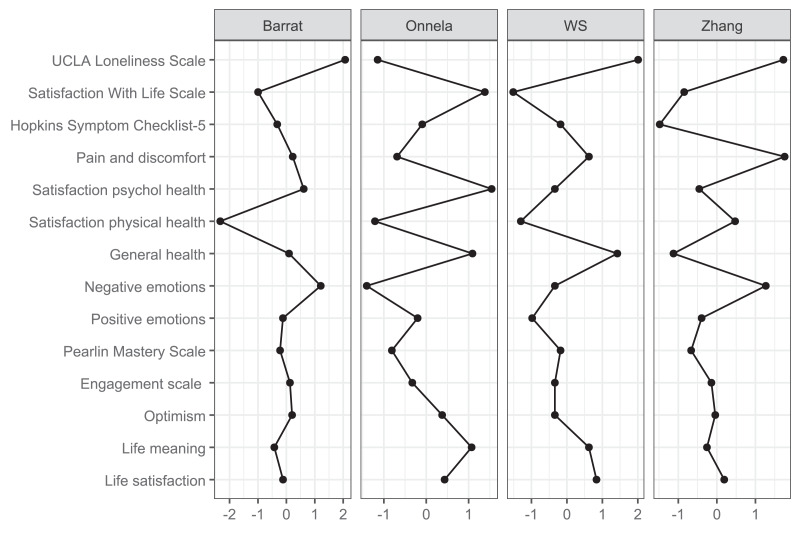
Clustering plot.

The network analysis revealed a structured constellation of relationships among the 14 constructs (Figs. 1–3, Supplement 1). The resulting network was moderately dense, indicating a coherent but differentiated structure across psychological, physical, and social domains. The Satisfaction with life scale emerged as the most topologically central node, with high closeness (1.872), strength (0.445), and moderately positive expected influence (0.165). Satisfaction with psychological health showed the highest closeness (1.838) and acted as an integrative variable across affective and evaluative domains despite a slightly negative expected influence (−0.500). Life satisfaction had fewer connections (strength = −0.071) but a high expected influence (0.825), indicating meaningful contributions to well-being *via* fewer but consistently positive links. Positive emotions and Life meaning further supported the well-being cluster. In contrast, Engagement and Optimism, though not strongly connected, exhibited positive expected influence (0.988 and 1.267, respectively), suggesting a supportive role.

Hopkins Symptom Checklist-5 stood out for its high strength (1.715), betweenness (1.694), and closeness (0.765), marking it as a key bridge between distress-related constructs and subjective well-being. Negative emotions were moderately connected to both distress and satisfaction variables, reinforcing their role in the emotional dimension of HRQoL. UCLA loneliness scale showed low global centrality (closeness = −1.272, strength = −1.827) but high local clustering (*e.g.*, Zhang = 1.309), suggesting that loneliness formed a dense but structurally independent subdomain.

Satisfaction with physical health, Pain and discomfort, and General Health were located at the network’s periphery. These variables exhibited low strength and closeness (*e.g.*, node 12: strength = −0.023; expected influence = −1.453) but high local clustering (*e.g.*, node 8: Zhang = 1.756), indicating a cohesive but isolated somatic subnetwork.

Finally, the Pearlin mastery scale displayed moderate centrality and positive influence, connecting to distress and resource indicators. Alongside optimism and engagement, it contributed to a diffuse resilience cluster linked indirectly to the central nodes.

Bootstrapped network stability analyses were conducted to evaluate model robustness (Supplement 1). The edge stability plot showed high consistency between bootstrap means and original estimates for the strongest positive and negative connections, particularly those involving Hopkins Symptom Checklist-5, Satisfaction with life scale, and Life satisfaction. However, many weaker edges showed considerable variation across bootstraps, suggesting these associations are less reliably estimated and should be interpreted cautiously. The case-dropping centrality stability analysis indicated that strength centrality exhibited the highest and most consistent stability (average correlation ∼0.35–0.45). In contrast, betweenness and closeness showed lower but relatively flat correlations across subsamples. This pattern likely reflects the stabilising effect of the large sample size and the dense interconnectivity among outcomes.

### Sensitivity analysis

Participants were divided into three language-based subsamples: Bokmål (*n* = 13,872), Nynorsk (*n* = 555), and English (*n* = 721). The mean age was 49.9 years (SD = 17.3) in Bokmål, 51.9 years (SD = 16.9) in Nynorsk, and 38.6 years (SD = 13.3) in English. The proportion of women was 52.3%, 48.1%, and 42.9%, respectively, and the proportion of first-generation immigrants was 9.1%, 2.5%, and 73.0%. Although there were some minor differences in health and QoL across the subsamples, with the English group tending to score somewhat poorer overall, the general pattern of psychometric results indicated good agreement with a few subtle variations. This suggests that the measures’ performances were stable across languages (e-Tables S4–S9 and e-Figs. S3–S5, Supplement 1).

## Discussion

Overall, the NQoLS measures demonstrated acceptable reliability and validity, supporting their use in public health surveillance of SRH and QoL. Sensitivity analysis suggests that the measures’ performances were stable across languages. While most measures performed well, a few of them may benefit from minor refinements to improve model fit or enhance sensitivity. Network analysis highlighted a core of psychological health and satisfaction with life, whereas physical health outcomes were weakly connected and assumed a more peripheral role.

### Psychometric analysis

The single- and multi-item scales used in the NQoLS demonstrated generally robust properties, with acceptable score distributions and satisfactory psychometric performance. However, pronounced ceiling effects were observed for the single-item measures General health and Pain and discomfort. These effects may partly reflect genuinely high levels of well-being and low pain prevalence within the general population. Alternatively, they may also arise from measurement constraints, such as a limited number of response categories and a restricted conceptual scope, which could reduce sensitivity at the upper end of the scale. Future public health studies addressing General health, pain and discomfort may therefore benefit from employing multi-item or condition-specific instruments when very high measurement precision is required (Norekvål et al., 2025). Confirmatory factor analyses supported the structural validity of the multi-item scales, although a few measures (*e.g.*, Pearlin mastery scale, and Negative emotions) showed elevated RMSEA values that required minor model modifications. However, RMSEA values remained slightly elevated for some scales, but this was not of major concern. As RMSEA may be biased under these conditions, greater weight was placed on the other indices, which indicated excellent fit and supported the conclusion that the overall model fit was well within acceptable limits (Shi, Maydeu-Olivares & Rosseel, 2020). Taken together, these findings suggest that the NQoLS multi-item scales provide a reliable assessment of SRH and QoL. Nonetheless, targeted refinements may further improve model fit for selected measures.

### Network analysis

Network analyses provided insights into how SRH and QoL outcomes were interlinked. Three measures emerged as especially central: the Satisfaction with life scale, the Hopkins Symptom Checklist-5, and Satisfaction with psychological health. These constructs bridged affective indicators (positive and negative emotions) with overall life evaluations, underscoring the pivotal role of psychological health in shaping wellbeing. While the Satisfaction with life scale anchored the central cluster, Satisfaction with psychological health was topologically closest to all other nodes, reinforcing its integrative function. The Hopkins Symptom Checklist-5 served as a bridge between wellbeing and distress, supporting models of mental health that emphasise measuring both symptoms and strengths. Life meaning and Positive emotions added further conceptual coherence to this cluster. Additionally, Life satisfaction—while less connected, had a strong indirect influence, indicating that global well-being evaluations can influenced by a few but high-quality connections. Psychological variables like Optimism, Engagement, and Mastery did not hold structurally central positions but consistently showed positive expected influence. Their role as facilitators of wellbeing aligns with theories of resilience and stress-buffering, suggesting that they may amplify or safeguard psychological functioning without directly anchoring the system.

The UCLA loneliness scale displayed a distinct structural profile. Despite having low global connectivity, its high clustering indicates that loneliness forms a distinct emotional domain with indirect links to broader wellbeing. These results align with recent research framing loneliness as a unique psychosocial determinant with important health implications (Klein et al., 2021). In contrast, physical health variables—Satisfaction with physical health, Pain and discomfort, and General health—occupied peripheral network positions. Although general health is often conceptualised as a summary indicator, it was weakly integrated with the network. This finding is consistent with previous studies suggesting that general health may be associated more with physical rather than psychological health (Andersen et al., 2022). While these variables formed a locally cohesive subnetwork, their limited reach into the broader system indicates that the current structure of the NQoLS may underrepresent somatic domains (Pettersen & Engvik, 2022). Overall, the network supports a multidimensional model in which psychological well-being and satisfaction with life form a densely integrated core, while distress, loneliness, physical complaints, and psychological resources occupy distinct yet interconnected peripheries. These results suggest that public health assessments and interventions should consider global well-being and the specific pathways through which emotional, somatic, and social experiences influence perceived health.

According to the Wilson and Cleary model, health outcomes form a pathway from biological variables and symptom status to broader domains of functioning, perceived health, and overall QoL (Wilson & Cleary, 1995). Our network findings partly support this idea, illustrating how psychological health indicators (*e.g.*, Hopkins Symptom Checklist-5) can bridge emotional functioning and global life satisfaction. Notably, general health—a pivotal construct in Wilson and Cleary’s hierarchy—exhibited relatively low connectivity. This has implications that merit further consideration. For example, the weak integration of general health in the network highlights the possible limitation of relying on this item in population surveys, particularly when complex health processes are involved. It also raises questions about how individuals weigh different aspects of SRH and QoL. The central positioning of psychological variables suggests that perceptions of SRH and QoL may be shaped more by emotional and cognitive states than by simple measures of physical health, as in this study. This highlights the importance of including subjective well-being and symptom burden, as well as more robust and comprehensive measures of physical health, in future research.

### Strengths and limitations of the NQoLS

A strength of the NQoLS is its broad scope, encompassing a wide range of variables. In addition to measuring SRH and QoL directly, it includes many factors that may influence these outcomes. This extensive coverage provides a strong foundation for population-level monitoring, enables the identification of vulnerable subgroups, and supports the design of targeted interventions to reduce health inequalities. However, the lack of alignment between the NQoLS and other major Norwegian cohort studies such as the HUNT Study and the TromsøStudy. represents a structural limitation. Using different SRH and QoL instruments across public health studies restricts comparability across cohorts and reduces the potential for longitudinal and cumulative analyses (Kaplan & Hays, 2022). Although user involvement was included in developing the NQoLS, harmonisation with established national surveys was not prioritised. Future iterations would benefit from incorporating a core set of indicators already used in large, longstanding studies, improving comparability and enabling integration across cohorts. Finally, although an expert group with relevant linguistic and cultural expertise designed and prepared the survey in three languages, the original report provides little information on this process (Nes, Hansen & Barstad, 2018). This may be considered another limitation of the NQoLS that readers should bear in mind when interpreting the results.

### Strengths and limitations of the current study

The current study enhances the NQoLS by integrating psychometric evaluation with network analysis, applied to a large and diverse sample. This approach offers a map of how SRH and QoL outcomes interrelate in a general population. The response rate was acceptable for studies of this, with minimal missing data. Nonetheless, several limitations should be acknowledged. The cross-sectional design prevents causal inference, and despite weighting, some demographic bias likely remains, particularly underrepresentation of older and less-educated individuals. It is also important to acknowledge that selection bias is inevitable in studies of this kind. Health problems and life difficulties may lead some individuals to refrain from participation, which means that the results are likely skewed toward better outcomes compared to what would have been observed if all eligible participants had taken part. Moreover, while the network structure was stable for strong associations, weaker edges showed more variability across bootstrap samples and should be interpreted cautiously. Thus, future research should aim to replicate these results and employ alternative methodological approaches to more robustly evaluate network patterns and their stability.

## Conclusions

The NQoLS demonstrated acceptable reliability and validity in capturing SRH and QoL across a broad and heterogeneous population. While overall psychometric performance was sound, some measures may benefit from further refinement to enhance sensitivity and model fit. The network analysis revealed a central core dominated by psychological health and life satisfaction, while physical health outcomes were positioned more peripherally within the overall structure. The findings point to two primary considerations for policymakers and researchers. First, psychological health and life satisfaction appear central to how the Norwegian population perceives overall health and wellbeing. Therefore, public health strategies aiming to improve QoL may achieve a broader impact by targeting psychological wellbeing as a key lever. Second, the network’s peripheral clustering of physical health measures suggests that the NQoLS could benefit from expanded assessments of SRH. Established instruments like PROMIS-29 (Garratt et al., 2021) or RAND-36 (Andersen et al., 2022; Hays & Morales, 2001) may offer more robust and nuanced evaluations of physical functioning and symptoms, aligning the survey more closely with integrative health frameworks.

##  Supplemental Information

10.7717/peerj.20529/supp-1 Supplemental Information 1 Supplemental figures and tables

## Additional Information and Declarations

### Data Availability

The following information was supplied regarding data availability: The data is available at: Statistics Norway. (2023). Quality of Life Survey 2022 (Version 1) [Quality of Life Survey 2022]. Sikt - Norwegian Agency for Shared Services in Education and Research. https://doi.org/10.18712/NSD-NSD3106-V1.
