# Supplementary material for: Mapping quality of life in Norway: psychometric evaluation and network analysis of 15,148 responses from a public health study
Source: PeerJ. 2026 Jan 13;14:e20529. doi: 10.7717/peerj.20529 (PMC12810394; doi:10.7717/peerj.20529)
Supplement: Supplemental Information 1 [file peerj-14-20529-s001.docx]

# **Supplementary Online Content** John Roger Andersen, Tone Nygaard Flølo, Kari Hanne Gjeilo, Käthe Meyer, Tone Merete Norekvål, Gudrun Rohde. Mapping quality of life in Norway: Psychometric evaluation and network analysis of 15,148 responses from a public health study

**Supplementary results for the pooled network analysis**

**e-Table 1.** Summary of Network **e-Table 2.** Centrality measures per variable  **e-Table 3.** Clustering measures per variable **e-Figure 1.** Bootstrap analysis with 1000 repetitions for edge stability. **e-Figure 2.** Bootstrap analysis with 1000 repetitions for central stability.

**Supplementary results for stratified analysis according to language**

**e-Table 4.** Descriptive statistics and psychometric properties of single-item measures included in the NQoLS for the sub-sample using the Norwegian language Bokmål (n = 13,872).

**e-Table 5.** Descriptive statistics and psychometric properties of multi-item scales included in the NQoLS for the sub-sample using the Norwegian language Bokmål (n = 13,872).

**e-Table 6.** Descriptive statistics and psychometric properties of single-item measures included in the NQoLS for the sub-sample using the Norwegian language Nynorsk (n = 555).

**e-Table 7.** Descriptive statistics and psychometric properties of multi-item scales included in the NQoLS for the sub-sample using the Norwegian language Nynorsk (n = 555).

**e-Table 8.** Descriptive statistics and psychometric properties of single-item measures included in the NQoLS for the sub-sample using English (n = 721).

**e-Table 9.** Descriptive statistics and psychometric properties of multi-item scales included in the NQoLS for the sub-sample using English (n = 721).

**e-Figure 3.** Pearson correlations and a heatmap for the sub-sample using the Norwegian language Bokmål (n = 13,872).

**e-Figure 4.** Pearson correlations and a heatmap for the sub-sample using the Norwegian language Nynorsk (n = 555).

**e-Figure 5.** Pearson correlations and a heatmap for the sub-sample using English (n = 721).

| **e-Table 1.** Summary of Network | | | | | |
| --- | --- | --- | --- | --- | --- |
| Number of nodes | | Number of non-zero edges | | Sparsity | |
| 14 |  | 75 / 91 |  | 0.176 |  |
|  | | | | | |

| **e-Table 2.** Centrality measures per variable | | | | | | | | | |
| --- | --- | --- | --- | --- | --- | --- | --- | --- | --- |
|  | | Network | | | | | | | |
| Variable | | Betweenness | | Closeness | | Strength | | Expected influence | |
| Engagement scale |  | -0.887 |  | -0.417 |  | -0.742 |  | 0.988 |  |
| General health |  | 0.887 |  | -0.590 |  | 1.095 |  | -1.921 |  |
| Hopkins Symptom Checklist-5 |  | 1.694 |  | 0.765 |  | 1.715 |  | 0.040 |  |
| Life meaning |  | -0.242 |  | 0.380 |  | 1.061 |  | 1.375 |  |
| Life satisfaction |  | -0.726 |  | 0.452 |  | -0.071 |  | 0.825 |  |
| Negative emotions |  | -1.049 |  | -0.772 |  | -0.881 |  | -0.037 |  |
| Optimism |  | 0.081 |  | -0.207 |  | 0.184 |  | 1.267 |  |
| Pain and discomfort |  | -1.049 |  | -1.243 |  | -1.010 |  | -7.649×10^-4^ |  |
| Pearlin mastery scale |  | -0.081 |  | -0.557 |  | -0.820 |  | -0.766 |  |
| Positive emotions |  | -0.242 |  | 0.384 |  | -0.176 |  | 0.776 |  |
| Satisfaction physical health |  | -0.081 |  | -0.633 |  | -0.023 |  | -1.453 |  |
| Satisfaction psychol health |  | 2.017 |  | 1.838 |  | 1.052 |  | -0.500 |  |
| Satisfaction with life scale |  | 0.726 |  | 1.872 |  | 0.445 |  | 0.165 |  |
| UCLA loneliness scale |  | -1.049 |  | -1.273 |  | -1.827 |  | -0.758 |  |
|  | | | | | | | | | |

| **e-Table 3.** Clustering measures per variable | | | | | | | | | |
| --- | --- | --- | --- | --- | --- | --- | --- | --- | --- |
|  | | Network | | | | | | | |
| Variable | | Barrat | | Onnela | | WS | | Zhang | |
| Hopkins Symptom Checklist-5 |  | -0.323 |  | -0.096 |  | -0.179 |  | -1.481 |  |
| Satisfaction with life scale |  | -0.996 |  | 1.382 |  | -1.514 |  | -0.847 |  |
| UCLA loneliness scale |  | 2.068 |  | -1.148 |  | 2.010 |  | 1.724 |  |
| Life satisfaction |  | -0.118 |  | 0.433 |  | 0.835 |  | 0.191 |  |
| Life meaning |  | -0.425 |  | 1.070 |  | 0.622 |  | -0.257 |  |
| Optimism |  | 0.201 |  | 0.374 |  | -0.339 |  | -0.042 |  |
| Engagement scale |  | 0.126 |  | -0.330 |  | -0.339 |  | -0.142 |  |
| Pearlin mastery scale |  | -0.222 |  | -0.812 |  | -0.179 |  | -0.667 |  |
| Positive emotions |  | -0.121 |  | -0.204 |  | -0.980 |  | -0.395 |  |
| Negative emotions |  | 1.210 |  | -1.404 |  | -0.339 |  | 1.268 |  |
| General health |  | 0.093 |  | 1.091 |  | 1.422 |  | -1.125 |  |
| Satisfaction physical health |  | -2.327 |  | -1.211 |  | -1.300 |  | 0.473 |  |
| Satisfaction psychol health |  | 0.611 |  | 1.542 |  | -0.339 |  | -0.456 |  |
| Pain and discomfort |  | 0.223 |  | -0.689 |  | 0.622 |  | 1.757 |  |
|  | | | | | | | | | |

**
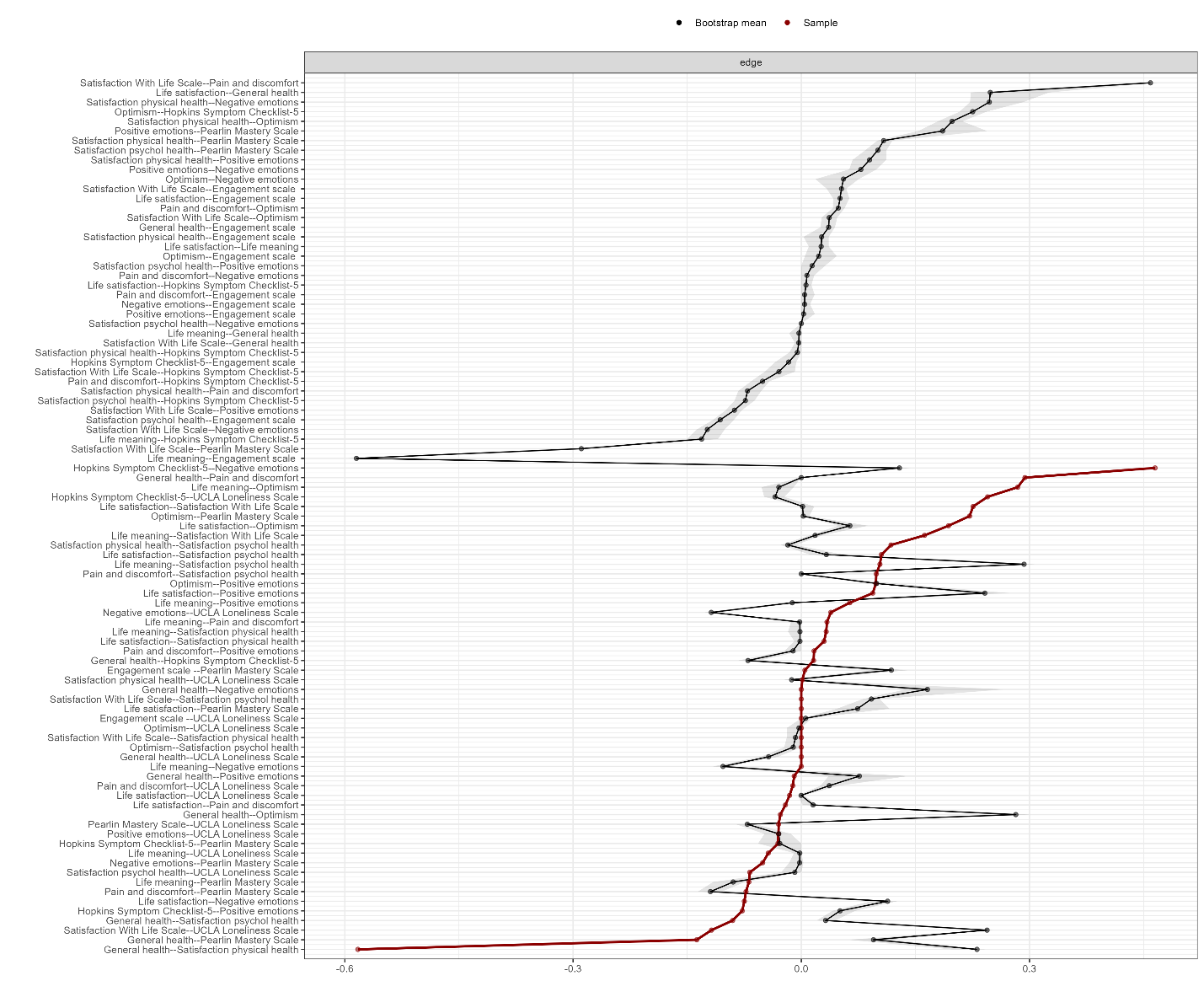
**

**e-Figure 1. Bootstrap analysis with 1000 repetitions for edge stability.**


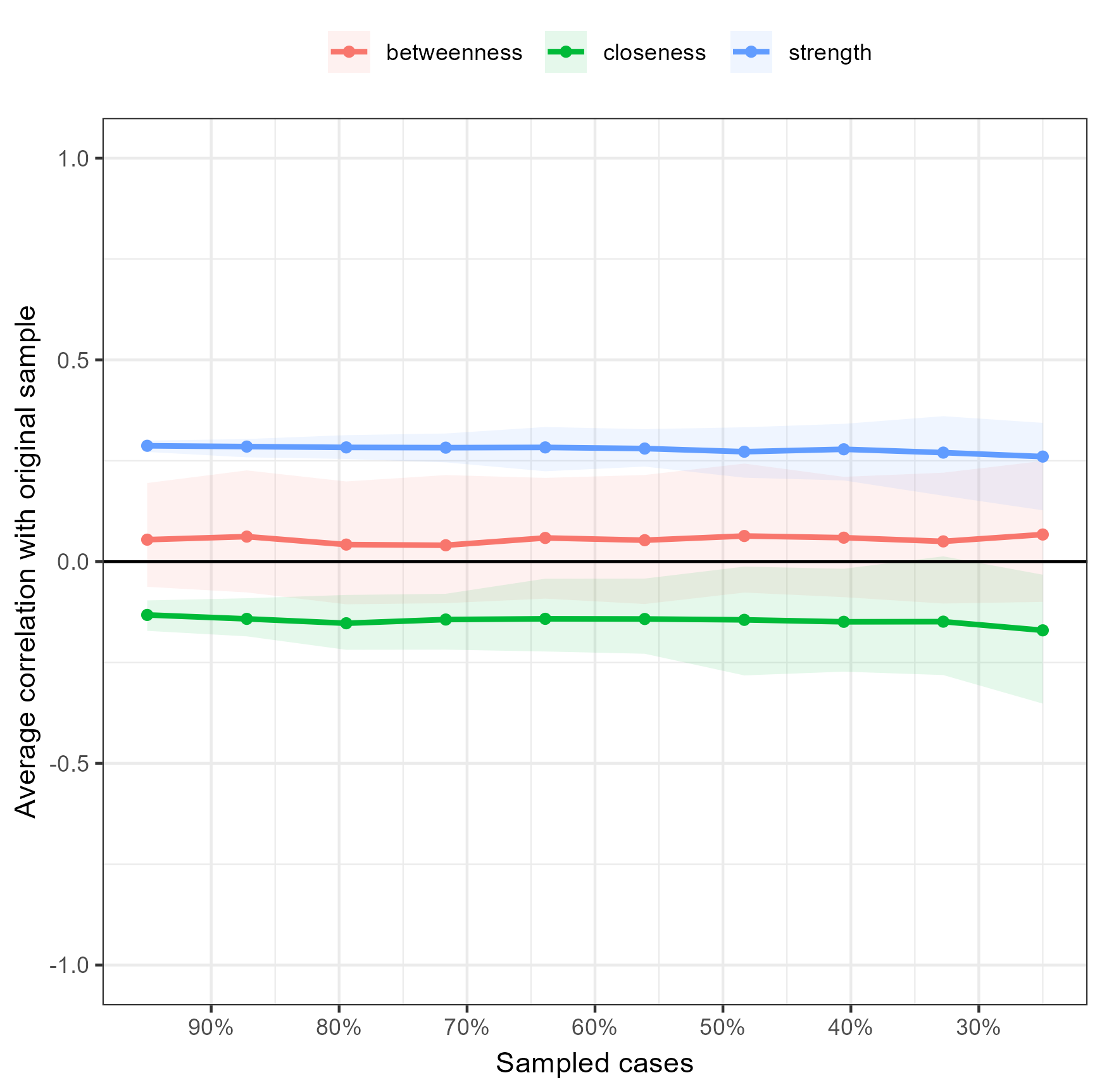


**e-Figure 2. Bootstrap analysis with 1000 repetitions for central stability.**

**e-Table 4.** Descriptive statistics and psychometric properties of single-item measures included in the NQoLS for the sub-sample using the Norwegian language Bokmål (n = 13,872).

| Features | Life satisfaction | Life meaning | General health | Satisfaction with physical health | Pain and discomfort | Satisfaction with psychological health | Optimism |
| --- | --- | --- | --- | --- | --- | --- | --- |
| Score | 0–10, higher score is better | 0–10, higher score is better | 1–5, lower score is better | 0–10, higher score is better | 1-–5, lower score is better | 0–10, higher score is better | 0-–10, higher score is better |
| Score range | 0–10 | 0–10 | 1–5 | 0–10 | 1–5 | 0–10 | 0–10 |
| Missing data,  % | .09 | .18 | .04 | .06 | .07 | .08 | 1.30 |
| Mean ± SD | 6.86 ± 2.22 | 7.07 ± 2.20 | 2.28 ± .93 | 6.32 ± 2.29 | 2.08 ± .94 | 7.08 ± 2.46 | 7.36 ± 2.09 |
| Median | 7 | 7 | 2 | 7 | 2 | 8 | 8 |
| Skewness | -.68 | -.85 | .68 | -.61 | .77 | -.89 | -1.03 |
| Kurtosis | .08 | .52 | .13 | .03 | .28 | .21 | 1.14 |
| % Floor | 1.1 | 1.1 | 1.7 | 2.2 | 1.5 | 2.0 | 1.0 |
| % Ceiling | 11.9 | 13.7 | 17.9 | 6.5 | 28.9 | 16.7 | 15.1 |

### Note: Floor effect = worst possible score, and ceiling effect = best possible score.

**e-Table 5.** Descriptive statistics and psychometric properties of multi-item scales included in the NQoLS for the sub-sample using the Norwegian language Bokmål (n = 13,872).

| Features | Satisfaction with life scale | Hopkins Symptom Checklist-5 | Positive emotions | Negative emotions | Engagement scale | Pearlin Mastery Scale | UCLA loneliness scale |
| --- | --- | --- | --- | --- | --- | --- | --- |
| Score | 1–7, higher score is better | 1–4, lower score is better | 0–10, higher score is better | 0–10, lower score is better | 0–10, higher score is better | 1–5, higher score is better | 1–4, lower score is better |
| Missing data, % | .32 | .14 | 0.22 | .19 | 0.7 | .04 | .11 |
| Mean ± SD | 5.10 ± 1.33 | 1.65 ± 0.66 | 6.75 ± 1.89 | 3.44 ± 2.21 | 6.82 ± 1.87 | 3.52 ± .83 | 2.22 ± .80 |
| Median | 5.40 | 1.40 | 7 | 3 | 7 | 3.60 | 2 |
| Skewness | -.88 | 1.26 | -.57 | .44 | -.77 | -.30 | .28 |
| Kurtosis | .16 | 1.28 | .14 | -.43 | .59 | -.29 | -.62 |
| % Floor | .4 | .7 | .3 | .4 | .3 | .4 | 3.7 |
| % Ceiling | 3.9 | 24.0 | 4.6 | 7.8 | 3.3 | 4.1 | 11.9 |
| Cronbach’s alpha | .90 | .89 | .82 | .87 | .92 | .73 | .85 |
| RMSEA | .14/.03 | .10/.06 | .07/.00 | .18/.11 | NA | .23/.03 | NA |
| SRMR | .02/.00 | .03/.01 | .01/.00 | .04/.02 | NA | .08/.01 | NA |
| CFI | .99/1 | .99/1 | 1/1 | .98/.99 | NA | .89/1 | NA |
| TLI | .99/1 | .99/1 | .99/1 | .96/.99 | NA | .78/1 | NA |
| Modification indices | Item 4*5  (406.1) | Item c*d (240.4) | Item 1*7 (41.0) | 4*9  (552.5) |  | Item 4*5 (2197.5) |  |

Note: Floor effect = worst possible score, and ceiling effect = best possible score. RMSEA = root mean square error of approximation, SRMR = standardised root mean square residual, CFI = comparative fit index, TLI = Tucker-Lewis index. NA = not applicable.

**e-Table 6.** Descriptive statistics and psychometric properties of single-item measures included in the NQoLS for the sub-sample using the Norwegian language Nynorsk (n = 555).

| Features | Life satisfaction | Life meaning | General health | Satisfaction with physical health | Pain and discomfort | Satisfaction with psychological health | Optimism |
| --- | --- | --- | --- | --- | --- | --- | --- |
| Score | 0–10, higher score is better | 0–10, higher score is better | 1–5, lower score is better | 0–10, higher score is better | 1-–5, lower score is better | 0–10, higher score is better | 0-–10, higher score is better |
| Missing data, % | 0 | 0 | 0 | 0 | 0.18 | 0 | 2.52 |
| Mean ± SD | 7.06 ± 2.15 | 7.40 ± 2.03 | 2.24 ± .87 | 6.53 ± 2.20 | 1.95 ± 0.84 | 7.29 ± 2.25 | 7.54 ± 1.93 |
| Median | 8 | 8 | 2 | 7 | 2 | 8 | 8 |
| Skewness | -.77 | -.91 | .80 | -.58 | .87 | -.86 | -1.05 |
| Kurtosis | .15 | .60 | .53 | -.06 | .80 | .23 | 1.17 |
| % Floor | .7 | .4 | 1.1 | 1.1 | .7 | .7 | .4 |
| % Ceiling | 12.3 | 14.2 | 15.7 | 7.0 | 31.0 | 17.5 | 14.4 |

### Note: Floor effect = worst possible score, and ceiling effect = best possible score.

**e-Table 7.** Descriptive statistics and psychometric properties of multi-item scales included in the NQoLS for the sub-sample using the Norwegian language Nynorsk (n = 555).

| Features | Satisfaction with life scale | Hopkins Symptom Checklist-5 | Positive emotions | Negative emotions | Engagement scale | Pearlin Mastery Scale | UCLA loneliness scale |
| --- | --- | --- | --- | --- | --- | --- | --- |
| Score | 1–7, higher score is better | 1–4, lower score is better | 0–10, higher score is better | 0–10, lower score is better | 0–10, higher score is better | 1–5, higher score is better | 1–4, lower score is better |
| Missing data, % | .18 | 0 | 0 | .18 | 0 | 0 | 0 |
| Mean ± SD | 5.23 ± 1.24 | 1.51 ± .53 | 6.85 ± 1.84 | 3.16 ± 2.04 | 6.70 ± 1.77 | 3.44 ± .82 | 2.19 ± .75 |
| Median | 5.60 | 1.40 | 7 | 3 | 7 | 3.40 | 2 |
| Skewness | -.97 | 1.31 | -.64 | .42 | -.68 | -.22 | .23 |
| Kurtosis | .36 | 1.70 | .45 | -.63 | .34 | -.29 | -.51 |
| % Floor | .4 | 0 | 0.2 | 0 | .2 | .5 | 2.3 |
| % Ceiling | 2.0 | 27.6 | 5.0 | 7.6 | 3.1 | 4.3 | 11.5 |
| Cronbach’s alpha | .90 | .85 | .82 | .85 | .91 | .73 | .85 |
| RMSEA | .17/.07 | .07/.01 | .15/.02 | .16/.14 | NA | .21/.00 | NA |
| SRMR | .03/.01 | .03/0.01 | .02/.00 | .03/.02 | NA | .08/.01 | NA |
| CFI | .99/1 | 1/1 | .99/1 | .98/.99 | NA | .90/1 | NA |
| TLI | .98/1 | .99/1 | .97/1 | .97/.97 | NA | .80/1 | NA |
| Modification indices | Item 4*5 (22.3) | Item b*d (5.9) | Item 1*7 (8.7) | Item 4*9 (9.6) |  | Item 4*5 (84.3) |  |

Note: Floor effect = worst possible score, and ceiling effect = best possible score. RMSEA = root mean square error of approximation, SRMR = standardised root mean square residual, CFI = comparative fit index, TLI = Tucker-Lewis index. NA = not applicable.

**e-Table 8.** Descriptive statistics and psychometric properties of single-item measures included in the NQoLS for the sub-sample using English (n = 721).

| Features | Life satisfaction | Life meaning | General health | Satisfaction with physical health | Pain and discomfort | Satisfaction with psychological health | Optimism |
| --- | --- | --- | --- | --- | --- | --- | --- |
| Score | 0–10, higher score is better | 0–10, higher score is better | 1–5, lower score is better | 0–10, higher score is better | 1-–5, lower score is better | 0–10, higher score is better | 0-–10, higher score is better |
| Missing data, % | 0.14 | .69 | .14 | .28 | .41 | .41 | 1.11 |
| Mean ± SD | 6.78 ± 2.26 | 7.05 ± 2.24 | 2.26 ± .91 | 6.68 ± 2.31 | 1.92 ± .94 | 6.71 ± 2.61 | 7.56 ± 2.13 |
| Median | 7 | 7 | 2 | 7 | 2 | 7 | 8 |
| Skewness | -.67 | -.78 | .75 | -.87 | .88 | -.74 | -1.30 |
| Kurtosis | .31 | .52 | .47 | .54 | .39 | -.16 | 1.99 |
| % Floor | 1.8 | 1.4 | 1.9 | 2.8 | 1.4 | 2.9 | 1.4 |
| % Ceiling | 12.8 | 16.1 | 17.8 | 8.5 | 40.0 | 14.9 | 18.7 |

### Note: Floor effect = worst possible score, and ceiling effect = best possible score.

**e-Table 9.** Descriptive statistics and psychometric properties of multi-item scales included in the NQoLS for the sub-sample using English (n = 721).

| Features | Satisfaction with life scale | Hopkins Symptom Checklist-5 | Positive emotions | Negative emotions | Engagement scale | Pearlin Mastery Scale | UCLA loneliness scale |
| --- | --- | --- | --- | --- | --- | --- | --- |
| Score | 1–7, higher score is better | 1–4, lower score is better | 0–10, higher score is better | 0–10, lower score is better | 0–10, higher score is better | 1–5, higher score is better | 1–4, lower score is better |
| Missing data, % | .42 | .69 | 1.11 | .55 | .28 | .55 | .69 |
| Mean ± SD | 4.71 ± 1.40 | 1.97 ± .71 | 6.48 ± 1.92 | 4.19 ± 2.40 | 6.81 ± 2.1 | 3.53 ± .75 | 2.55 ± .83 |
| Median | 5 | 1.80 | 7 | 4 | 7 | 3.60 | .2.67 |
| Skewness | -.66 | .63 | -.50 | .03 | -.73 | -.31 | -.08 |
| Kurtosis | -.21 | -.24 | .25 | -.87 | .47 | 0.01 | -.24 |
| % Floor | 1.1 | 1.0 | .6 | .7 | .7 | .6 | 7.5 |
| % Ceiling | 2.9 | 10.3 | 4.6 | 5.9 | 6.4 | 3.1 | 7.1 |
| Cronbach’s alpha | .90 | .86 | .75 | .88 | .92 | .78 | .82 |
| RMSEA | .12/.11 | .14/.10 | .13/.04 | .17/.14 | NA | .23/.00 | NA |
| SRMR | .02/.02 | .04/.00 | .03/0.01 | .03/.02 | NA | .07/.01 | NA |
| CFI | 1/1 | .98/.99 | .99/1 | .98/.99 | NA | .94/1 | NA |
| TLI | .99/.99 | .97/.98 | .97/1 | .96/.98 | NA | .88/1 | NA |
| Modification indices | Item 4*5 (7.3) | Item c*d  (21.3) | Item 1*7 (9.3) | Item 4*8  (13.5) |  | Item 4*5  (100.5) |  |

Note: Floor effect = worst possible score, and ceiling effect = best possible score. RMSEA = root mean square error of approximation, SRMR = standardised root mean square residual, CFI = comparative fit index, TLI = Tucker-Lewis index. NA = not applicable.


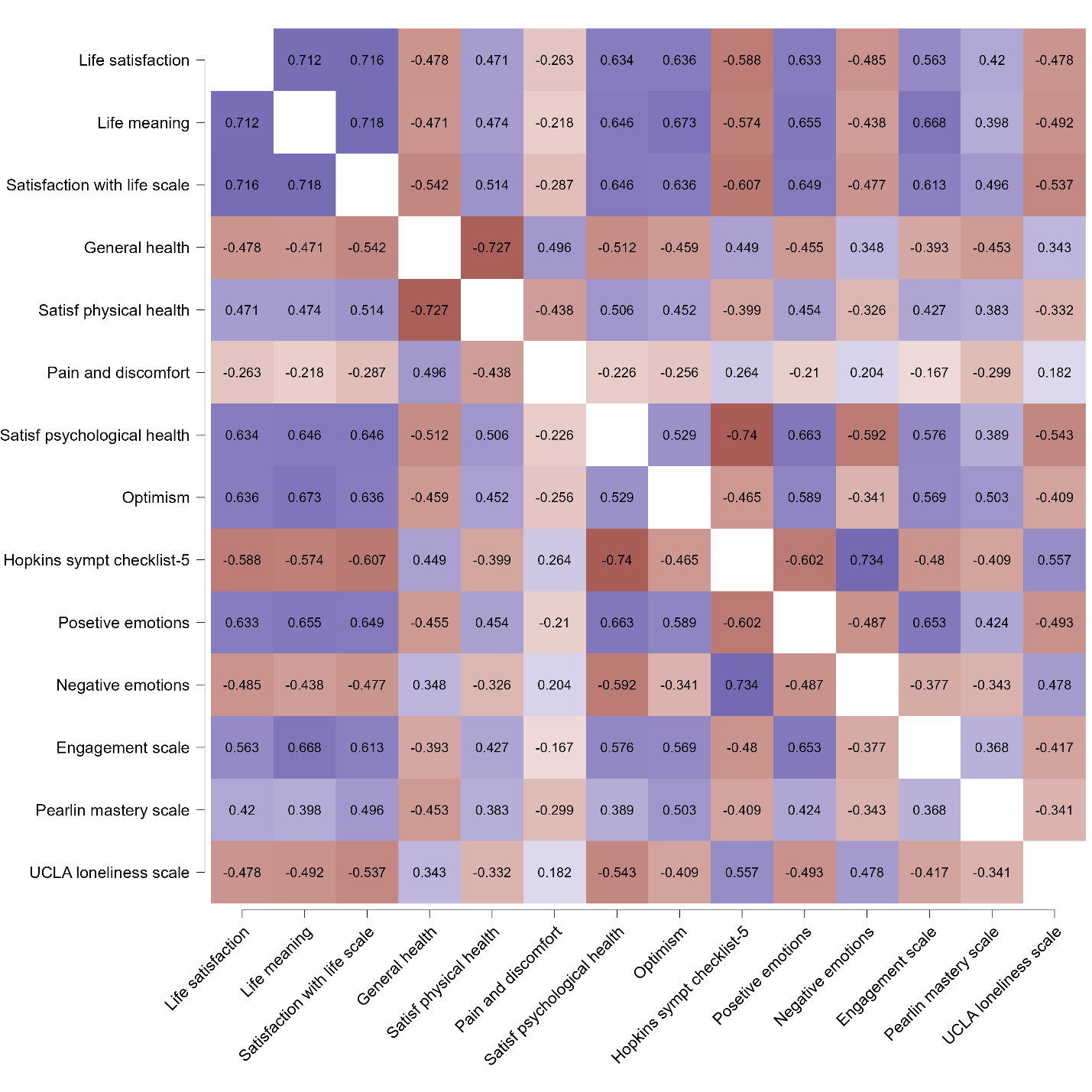


**e-Figure 3.** Pearson correlations and a heatmap for the sub-sample using the Norwegian language Bokmål (n = 13,872).


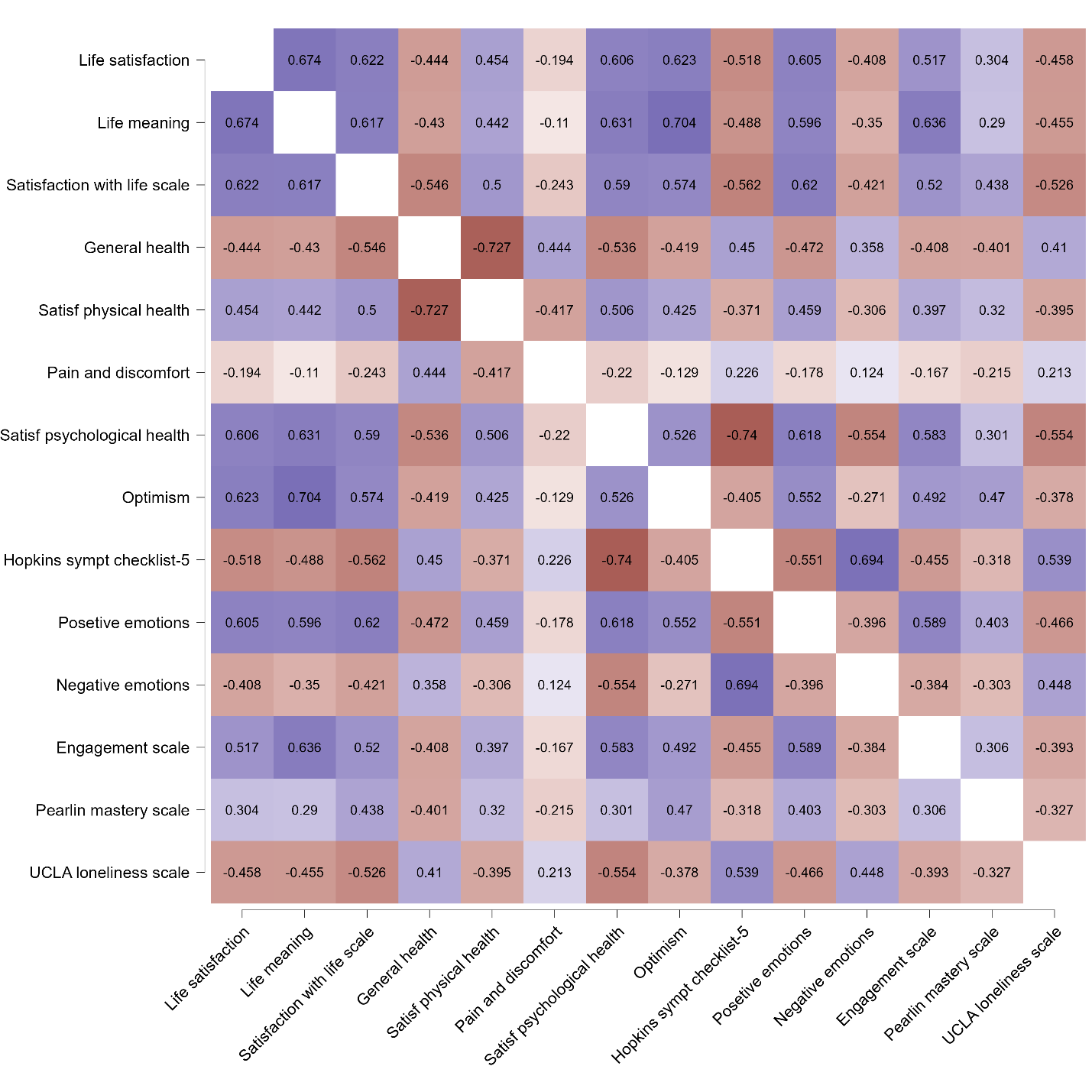


**e-Figure 4.** Pearson correlations and a heatmap for the sub-sample using the Norwegian language Nynorsk (n = 555).


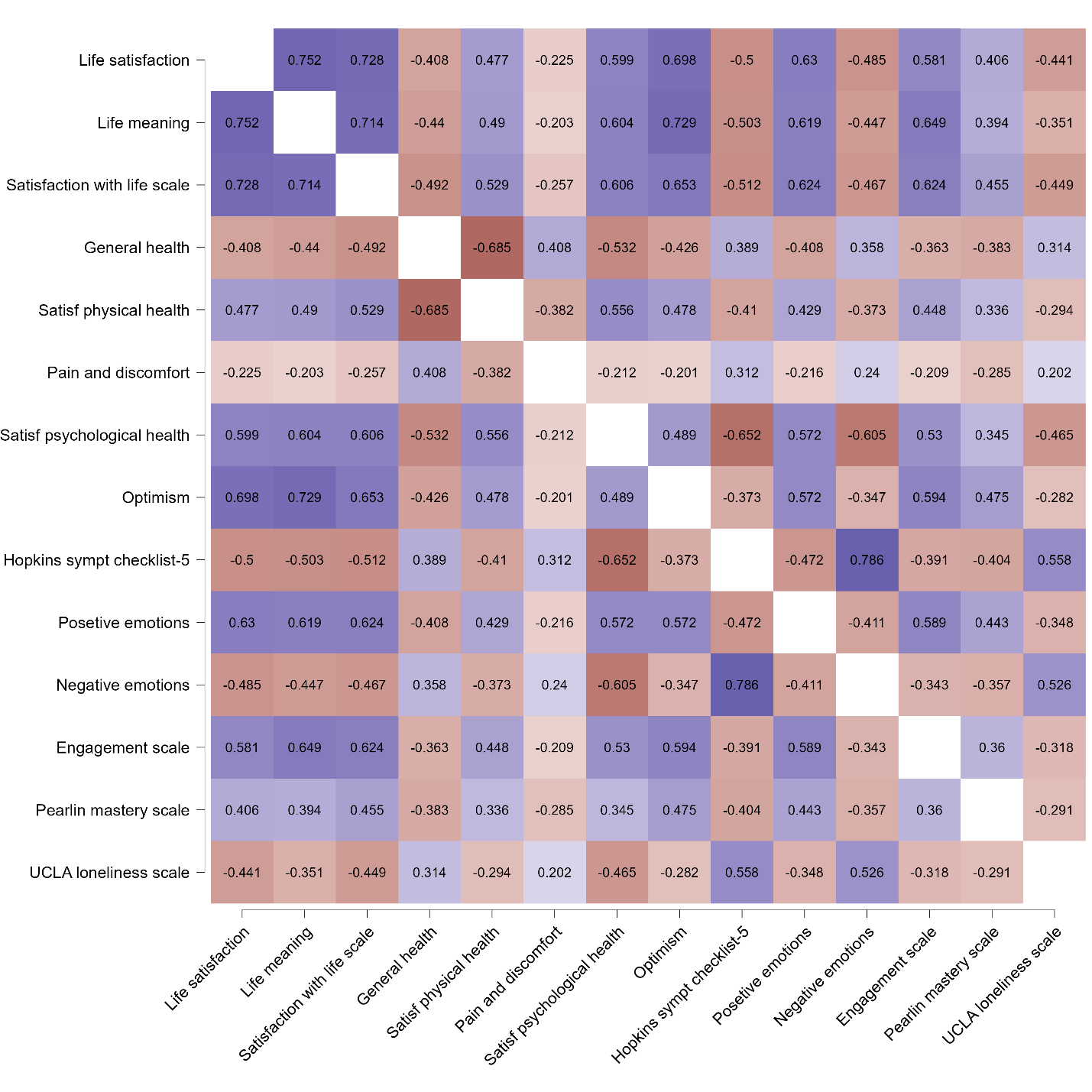


**e-Figure 5.** Pearson correlations and a heatmap for the sub-sample using English (n = 721).
